# Optimization of Bear Oil Extraction Process and Hair Growth Activity

**DOI:** 10.3390/molecules29061311

**Published:** 2024-03-15

**Authors:** Ziming Wang, Qiu Wang, Yingrui Jin, Kun Guo, Xiaoling Wang, Xueting Feng

**Affiliations:** College of Pharmacy, Southwest Minzu University, Chengdu 610041, China; 80300225@swun.edu.cn (Z.W.); 21900034@swun.edu.cn (Q.W.); jinyr111@163.com (Y.J.); guokun@swun.edu.cn (K.G.)

**Keywords:** bear fat, visual analysis, extraction process, compositional analysis, GC-MS, hair growth activity

## Abstract

According to ancient Chinese books, bear grease has the effects of strengthening muscles and bones, which is beneficial for weakness, but there is relatively little research on it. Thus, the extraction of it is beneficial for compensating for research in this area. In this study, a uniform experimental design method was used to optimize the extraction process of bear grease by enzymatic hydrolysis extraction, and the extraction rate can reach 81.89% under optimized extraction conditions. Furthermore, the components of bear grease obtained by this study were analyzed by GC-MS, and the results showed that ursolic oil was rich in unsaturated fatty acids (67.51%), which was higher than that of the traditional method (66.92%). The composition of bear grease extracted by the enzymatic method was also better than that extracted by the traditional method. In addition, bear grease obtained in this study had the obvious activity of promoting hair growth. The length, weight, and number of hair follicles in the depilation area of mice in the high-dose group were significantly different from those in the blank group (*p* < 0.01). This study optimized the extraction process of bear grease and conducted a preliminary analysis of its fatty acid composition, which is expected to provide some reference for the development of the medicinal value of bear grease.

## 1. Introduction

Bear fat is derived from the bear family animal black bear (*Selenarctos thibetanus G. Cuvier*). Bear fat is the fat oil of *Selenarctos thibetanus G. Cuvier*. The traditional extraction process is to take out the fat of artificially bred black bears after death, boil it, and filter off the oil residue to obtain bear fat oil. It is recorded in ancient books that bear grease can replenish deficiency; strengthen the muscles and bones; moisten the skin; treat wind paralysis, contracture of the tendons and veins, deficiency and emaciation, ringworm, white baldness, and polycarbonate sores; and, according to modern research, can also be used to prevent and control the efficacy of alopecia. The bear fat used in this experiment was obtained after the natural death of the bears.

At present, the extraction technology of animal and plant oils and fats is relatively mature. The traditional methods include boiling, pressing, alkaline hydrolysis, solvent extraction steaming, etc. The more advanced methods include enzymatic hydrolysis, membrane separation, supercritical extraction, and microwave-assisted extraction, etc. [1]. Enzymolysis is a technique of extracting fats and oils using enzymes as the extraction medium, and the choice of enzymes depends on the structure of the substrate and the composition of the cell wall. It was found that an enzyme-to-substrate ratio of 1% to 8%, a temperature of 40 to 55 °C, and a pH of 4 to 8 are typical conditions for enzyme extraction of oils and fats, and the specific operating conditions should be adjusted according to the type of enzyme [2]. The traditional method of oil extraction has problems such as a low extraction rate, affecting the quality of the product and causing environmental pollution [3,4,5,6].

Animal fats and oils are the earliest commonly consumed fats and oils by human beings, and there are many varieties of them, which are generally combined with proteins to generate lipoproteins or encapsulated proteins in animals. Enzymatic digestion can use proteases and other biological enzymes to degrade proteins, lipoproteins, and other complexes and promote the release of fats and oils without affecting the quality of the fats and oils [7]. Enzymatic digestion is currently more commonly used in domestic and international research; the extraction rate can reach more than 90%, and the lowest extraction rate can reach more than 70%. When extracting from animal fats and oils, the enzymes used are selective proteases, a situation related to the fact that animal fats and oils are mostly in the form of lipoproteins or protein-coated lipid vesicles. According to literature reports regarding aqueous enzyme extraction (AEE) of lard from pork fat, the lard obtained was superior to conventional extraction methods in terms of color, acid value, peroxide value, phospholipids, cholesterol, and oxidative stability [8]. In addition, the central combination design was used to optimize the extraction process of yellow mealworm oil, using alkaline protease and trypsin for compound hydrolysis, and the optimized oil extraction rate reached 78.51%. In conclusion, the present study proposes to use the enzymatic hydrolysis method for the extraction of bear grease.

To our knowledge, there are no studies on the extraction of bear grease by enzymatic digestion at home and abroad, so in this study, we proposed to extract bear grease by enzymatic digestion and optimize the process. For the optimization of the extraction process, the orthogonal method is mostly used at this stage for the experimental design, and it is difficult to arrange the orthogonal test when the number of factors or levels of factors to be examined in the test is very large in the practical application, so we choose the homogeneous design method to reduce the workload and obtain the experimental results with multiple levels, a wide range of data, and a large degree of confidence. For the analysis of the experimental results, we used the visual analysis (VA) method, which can intuitively reflect the optimal extraction process conditions within the study range [9].

## 2. Results and Discussion

### 2.1. Results of the Uniform Design of Experiments

The extraction rate of the bear grease by enzymatic method is shown in Table 1.

From Table 1, it can be seen that the results of the uniform design are not neatly comparable and cannot be analyzed by the ordinary VA act. In this experiment, the VA method was used to analyze them, and the experimental data were plotted with the investigation indexes into multiple 2.5-dimensional plots for a comprehensive evaluation to find out the optimal process conditions.

### 2.2. Visual Analysis

**Figure 1:**
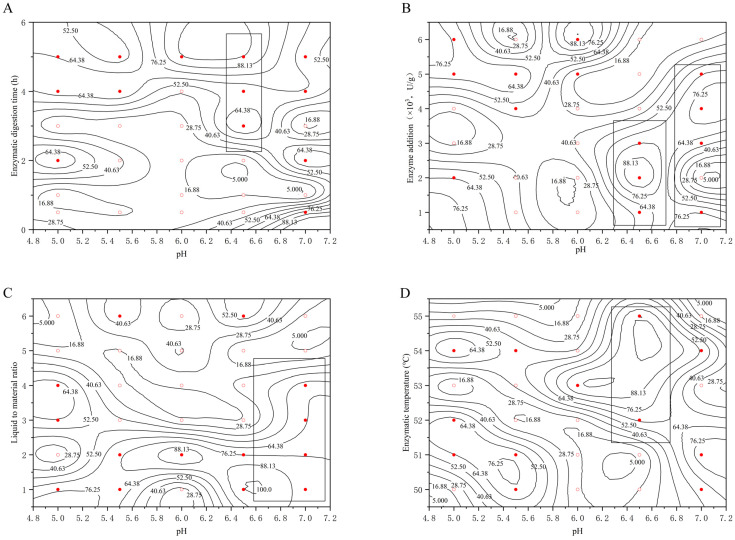
Effect of pH on extraction rate at different (**A**) enzyme digestion times; (**B**) enzyme additions; (**C**) liquid/feed ratios; (**D**) digestion temperatures. (○: the extraction rate η < 44.00, ●: the extraction rate η ≥ 44.00).

The values on the line in Figure 1 represent the extraction rate, and the points in the figure are divided into two categories according to the experimental results: the first category (○) represents the extraction rate η < 44.00, the second category (●) represents the extraction rate η ≥ 44.00, and 44.00 is the average value of the extraction rate. To study the effect of each factor on the extraction rate, the rectangular box is used to select the region with η ≥ 44.00, and the VA method is judged based on the longitudinal length of the rectangular box–penetration so that we can very intuitively observe the size of the probability that the optimization region exists in the figure.

#### 2.2.1. Effect of the pH on the Extraction Rate of Bear Grease

The effect of the pH on the extraction rate of bear grease under the conditions of different examined factors (enzyme digestion time, enzyme digestion temperature, liquid-to-material ratio, and enzyme addition) are shown in Figure 1.

As can be seen in Figure 1, the rectangular boxes were selected to show that the regions with η ≥ 44.00 were all located on the right side of the graph with good penetration. That means the pH in the interval of about 6.5–7 and the extraction rate of the bear grease was high and mainly influenced by pH. Therefore, the optimum pH of the extraction was between 6.5 and 7.

#### 2.2.2. Determination of Optimal Enzymatic Digestion Time

**Figure 2:**
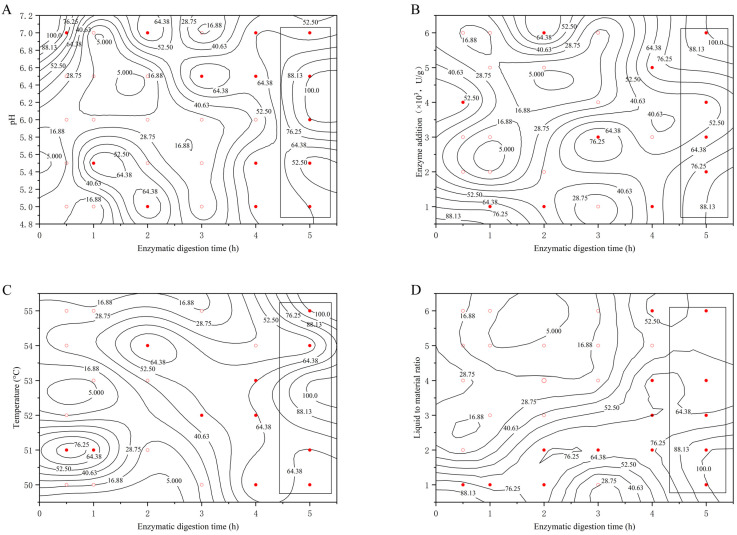
Influence of enzyme digestion time on extraction rate at different (**A**) pHs; (**B**) enzyme additions; (**C**) enzyme digestion temperatures; (**D**) liquid–feed ratios. (○: the extraction rate η < 44.00, ●: the extraction rate η ≥ 44.00).

Figure 2 plots the experimental data into a 2.5-dimensional graph with enzymatic digestion time as the horizontal coordinate and pH, enzymatic digestion temperature, enzymatic digestion time, and enzyme addition as the vertical coordinate, respectively, reflecting the effect of enzymatic digestion time on the extraction rate of bear grease under the conditions of different factors examined.

Comprehensively analyzed in Figure 2, the penetrations of the rectangular boxes were all deeper and the areas were more consistent. That is to say, when the extraction time was 4.5–5 h, no matter how the pH, enzyme digestion temperature, liquid–feed ratio, and enzyme addition changed, it did not have much effect on the extraction rate of bear grease. In conclusion, the effect of enzyme digestion time is more accurate, and the results can be judged that the optimal extraction time of bear fat can be set at 4.5–5 h.

#### 2.2.3. Determination of the Optimal Enzyme Digestion Temperature

**Figure 3:**
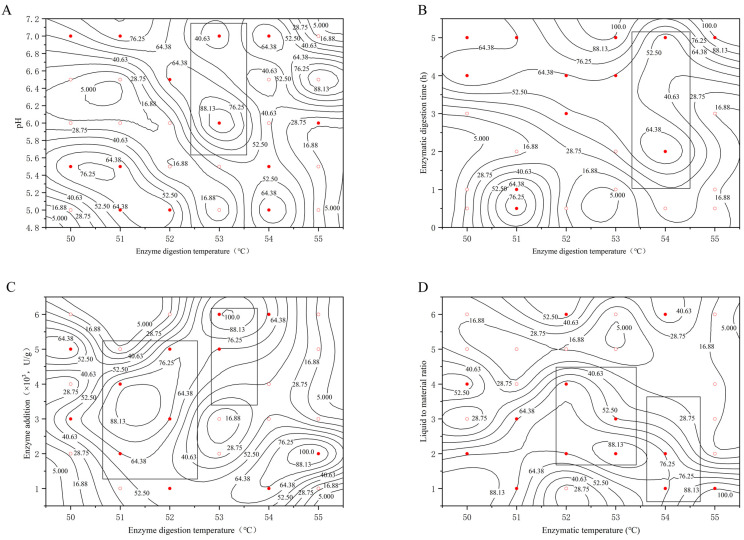
Effect of enzyme digestion temperature at different (**A**) pH; (**B**) enzyme digestion time; (**C**) enzyme additions; (**D**) liquid–feed ratios. (○: the extraction rate η < 44.00, ●: the extraction rate η ≥ 44.00).

Figure 3 plots the experimental data into a 2.5-dimensional graph with enzymatic temperature as the horizontal coordinate and pH, enzymatic time, liquid-to-material ratio, and enzyme addition as the vertical coordinate, respectively, reflecting the effect of enzymatic temperature on the extraction rate under the conditions of different factors examined.

As can be seen from the figure, the rectangular box that selects the region with η ≥ 44.00 has a more complex distribution on the graph. In Figure 3A, there are two regions crossed by the rectangular box: one, at a temperature of 52–53 °C, with a liquid-to-feed ratio between 2 and 4.5, and two, at a temperature of 53.5–54.5 °C, with a liquid-to-feed ratio of 1–3. Figure 3D also has two areas through the rectangular box, one is at the temperature of 50.5–52.5 °C, the enzyme addition between 1500~5000 U·g^−1^; the second is at the temperature of 53–54 °C, the enzyme addition between 3500–6000 U·g^−1^. Combining the four graphs Figure 3A–D, there are two possibilities for the optimal process range of enzyme digestion temperature, 50.5–52.5 °C and 53–54.5 °C.

The analysis of the four graphs shows that the effect of enzymatic digestion temperature on the extraction rate is more complicated. The reason for this situation may be, on the one hand, the activity of the protease used cannot be controlled, the optimal temperature used by the protease is between 50–55 °C, and its activity may not be completely consistent at different temperatures; on the other hand, the error generated during the experimental operation. Taken together, the optimal process temperature for extracting bear grease should be 52–54 °C.

#### 2.2.4. Determination of Optimal Liquid-Feed Ratio

**Figure 4:**
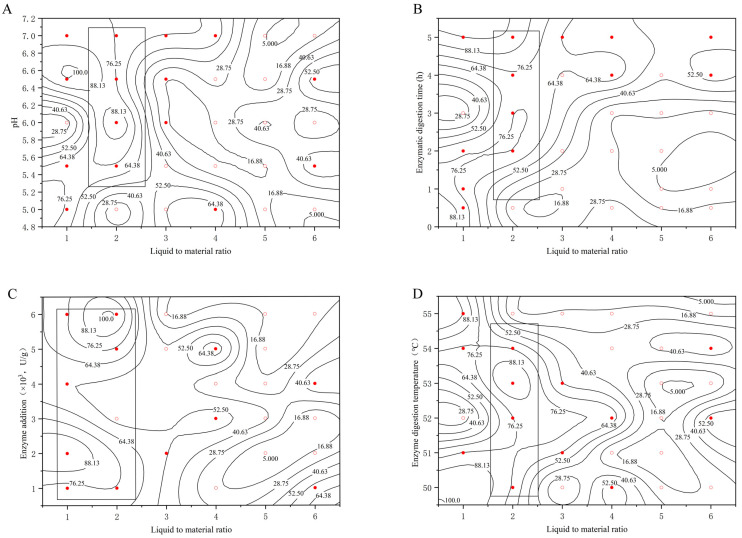
Influence of liquid–liquid ratio on the extraction rate at different (**A**) pHs; (**B**) enzyme digestion time; (**C**) enzyme addition amounts; (**D**) enzyme digestion temperatures. (○: the extraction rate η < 44.00, ●: the extraction rate η ≥ 44.00).

Figure 4 plots the experimental data into a 2.5-dimensional graph with the liquid–feed ratio as the horizontal coordinate and the pH, enzyme digestion time, enzyme digestion temperature, and enzyme addition as the vertical coordinate, respectively, reflecting the effect of the liquid–feed ratio on the extraction rate under the influence of the conditions of different investigated factors.

Combined with the above four graphs analysis, the liquid–feed ratio is in the range of 1–2, the penetration of the rectangular box is deeper, and it is located in the left part of the graph, almost from bottom to top penetration. This indicates that for the liquid–feed ratio in this interval, no matter how the other factors are varied, the extraction rate is always a large value, which concludes that the optimal range of liquid–feed ratios for the extraction of bear grease is 1:1–2:1.

#### 2.2.5. Determination of Optimal Enzyme Addition

**Figure 5:**
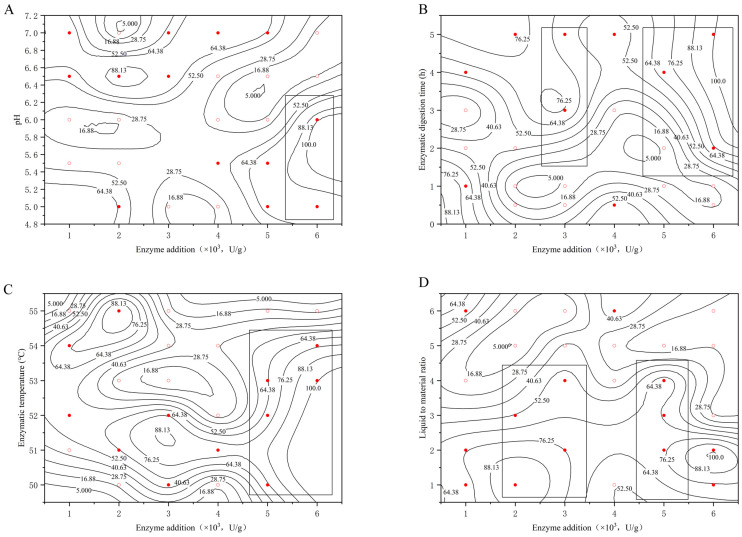
Effect of enzyme addition on the extraction rate at different (**A**) pHs; (**B**) digestion time; (**C**) digestion temperatures; (**D**) liquid–feed ratios. (○: the extraction rate η < 44.00, ●: the extraction rate η ≥ 44.00).

Figure 5 plots the experimental data into a 2.5-dimensional graph with the enzyme addition amount as the horizontal coordinate and pH, enzyme digestion time, enzyme digestion temperature, and liquid–feed ratio as the vertical coordinate, respectively, as a reflection of the effect of the enzyme addition amount on the extraction rate of bear grease under the conditions of different examined factors.

As can be seen in the four figures above, the rectangular boxes were selected to be more complex in the region of η ≥ 44.00%. In Figure 5C, there are rectangular boxes running through the enzyme additions in the ranges of 2500–3500 U·g^−1^ and 5000–6000 U·g^−1^, but there are hollow points in the range of 2500–3500 U·g^−1^ and the degree of penetration is slightly lower than that in the range of 5000–6000 U·g^−1^. In the Figure 5D graph, there are also two rectangular boxes in the range of 2000–3500 U·g^−1^ and 4500–5500 U·g^−1^, respectively, indicating that the effect of liquid material ratio on the enzymatic extraction of bear grease is more complex and no longer a single trend. However, it can be seen from the figure that the 2000–3500 U·g^−1^ rectangular box is located in the range of penetration that is a shallow and hollow point penetration, while the figure of the other rectangular box is located in the region of penetration that is better than the 2000–3500 U·g^−1^ rectangular box located in the region. Taken together, the four graphs show that the optimum process range for enzyme addition can be set at 5000–6000 U·g^−1^.

By taking pH, enzyme digestion temperature, enzyme digestion time, enzyme addition amount, and liquid–feed ratio as the factors to be examined, selecting the extraction rate of bear grease as the index of the experiment, using the uniform design method for the design of the experiment, and using the visual analysis method to analyze the experimental results, it was concluded that the optimal range of process for the extraction of bear grease was as follows: pH 6.5–7, extraction temperature 52–54 °C, liquid–feed ratio 2:1, enzyme digestion time 4.5–5 h, enzyme addition amount 5000–6000 U·g^−1^.

In the advantageous process interval, certain conditions were selected to perform the validation test: pH 6.5, enzyme digestion time 4.5 h, enzyme digestion temperature 53 °C, liquid-feed ratio 2:1, enzyme addition 5500 U·g^−1^; the extraction rate of bear fat was 81.89%, which was higher than the average value of 44.00% in the designed experiments.

#### 2.2.6. Discussion

Although the traditional extraction method is simple and low-cost, it still has a lot of disadvantages, such as large consumption of organic reagents, pollution of the environment, low quality of the extracted oils and fats, and low extraction rate [10]. According to the relevant literature, the extraction rate of the traditional process with high quality is generally located between 20% and 30% [11], while the enzymatic method can reach 70% to 90% [12]; in this study, the extraction rate could reach 81.89% after optimization. Moreover, the enzyme digestion method used in this study is characterized by mild conditions, high extraction rate, no pollution, and good performance compared with the traditional process.

## 3. GC-MS Results and Analysis

**Figure 6:**
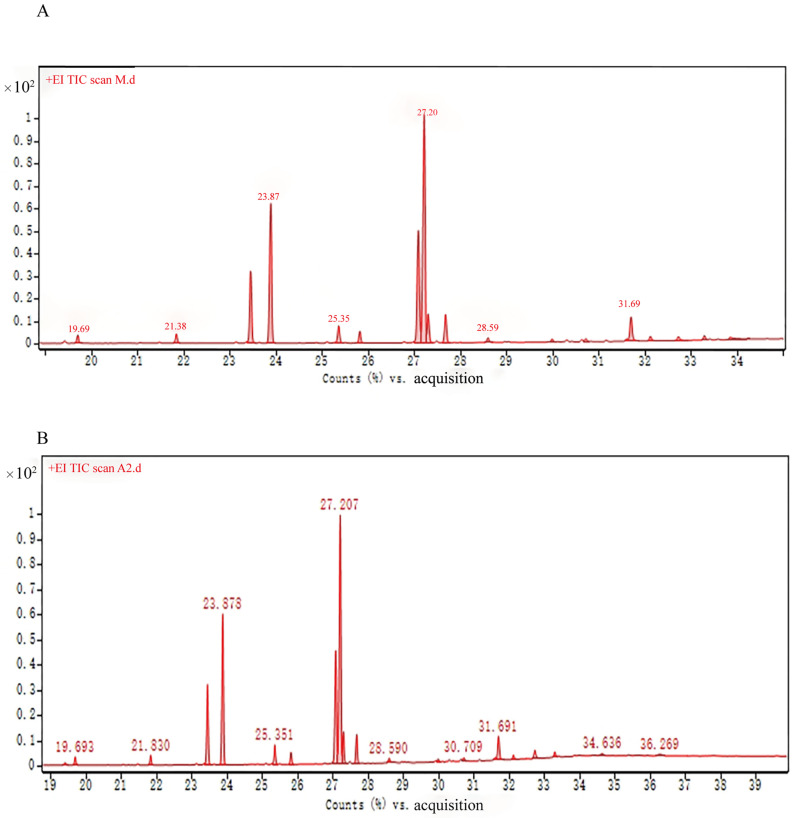
Fatty acid methyl ester total ionogram. (**A**) Bear grease obtained by enzymatic digestion; (**B**) bear grease obtained by traditional boiling method.

According to the GC-MS analysis conditions, the methyl esterification treatment solution of bear grease obtained by boiling method and the methyl esterification treatment solution of bear grease obtained by enzyme digestion method was analyzed, and the total ionogram of bear grease after methyl esterification was obtained, which is shown in Figure 6. Each chromatographic peak on the mass spectrometry diagram was compared with the standard spectrum of the spectral library, the chemical compositions were identified, and the relative contents of the compositions were calculated, which are shown in Table 2 and Table 3. The content of unsaturated fatty acids in bear grease obtained by boiling method was 66.92%, and the content of saturated fatty acids was 24.97%, while the content of unsaturated fatty acids in bear grease obtained by enzymatic digestion method was 67.51%, and the content of saturated fatty acids was 25.67%.

As can be seen from Table 2 and Table 3, the enzyme digestion method has more kinds of chemical components from bear fat compared with the boiling method, and the content of unsaturated fatty acids is also higher than that of bear fat obtained by the boiling method, which can show that the enzyme digestion method for the extraction of oil and grease has some advantages that the traditional boiling method does not have. Bear grease has a high content of unsaturated fatty acids, including linoleic acid, oleic acid, palmitic acid, and a small amount of myristic acid, stearic acid, arachidonic acid, myristoleucine, etc. Cetanoic acid is also known as palmitic acid, but it is also called hexadecanoic acid. Hexadecanoic acid, also known as palmitic acid, is a high-quality food emulsifier and fortifier, and its derivative isopropyl palmitate is a good emollient and is also the main ingredient in cosmetics; octadecenoic acid, also known as oleic acid, has strong penetration ability, has the efficacy of skincare, anti-wrinkle properties, preventing aging, etc., and is also used as an antistatic agent, lubricant, and emulsifier, etc. Octadecanoic acid, also known as stearic acid, is found in the plastics industry and the cosmetics industry. Octadecanoic acid is also called stearic acid, which is more common in plastics and cosmetics industries. All in all, through the chemical composition analysis, it can be concluded that bear oil has an efficacy that is worth further in-depth study.

The GC-MS analysis showed that bear grease contained more unsaturated fatty acids, with oleic acid, linoleic acid, stearic acid, palmitic acid, and arachidonic acid as the major ones. Recent studies have shown that polyunsaturated fatty acids (PUFA) have a variety of pharmacological effects such as promoting wound healing, lowering cholesterol, and eliminating inflammation, and it has been documented that linoleic acid may attenuate testosterone-induced signaling molecules and induce the growth of human hair follicle dermal papilla cells by activating Wnt/ß-catenin signaling. In this study, it is hoped that the fatty acid composition of bear grease can be analyzed to contribute to the subsequent development of the medicinal value of bear grease.

In conclusion, the bear grease extracted by the enzymatic method was better than that extracted by a traditional process in terms of fatty acid composition and unsaturated fatty acid content. In addition, the enzymatic extraction process is mild, and the degradation products produced will not react with the target extract, which can protect the effective extracted components and is in line with the current call for green, efficient, and safe products.

## 4. Results of Pharmacodynamic Experiments

**Figure 7:**
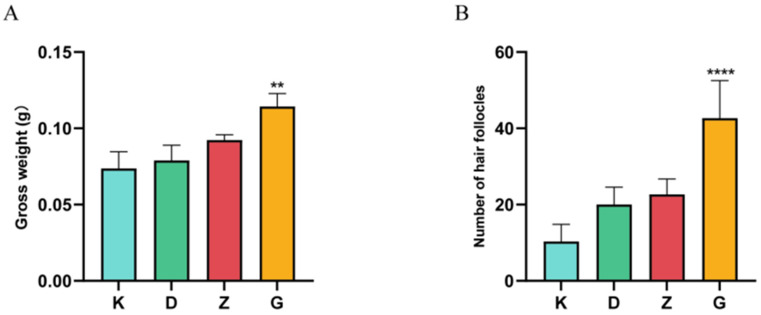
Effect of hair growth gel on body hair weight and number of hair follicles in hair removal area of mice. Gross weight (**A**); number of hair follicles (**B**); K-blank group, D-low dose group, Z-medium dose group, G-high dose group; ** *p* < 0.01 **** *p* < 0.001 (compared with blank group).

**Figure 8:**
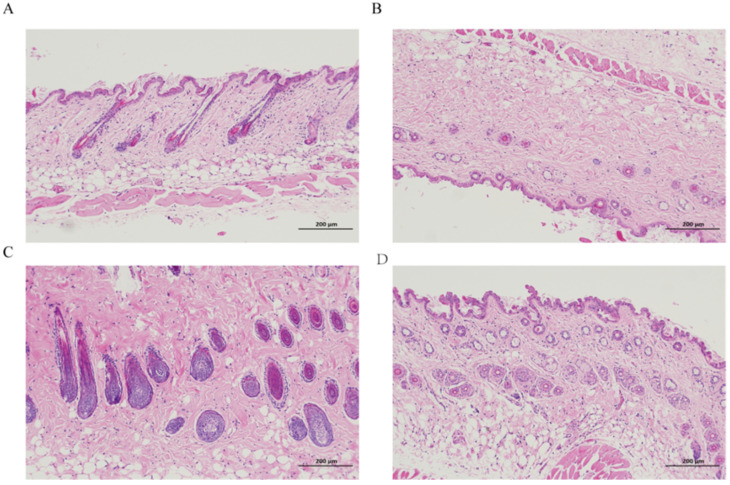
Tissue sections of mouse skin. (**A**) blank group, (**B**) low-dose group, (**C**) medium-dose group, (**D**) high-dose group.

The results showed that compared with the blank matrix, the hair regrowth gel increased the hair weight and the number of hair follicles of mice in all dose groups to different degrees, of which the mean value of hair weight and the mean value of the number of hair follicles in the high-dose group were significantly different from those in the blank matrix group (*p* < 0.01), as shown in Figure 7; from Figure 8, the number of hair follicles was as follows: high-dose group > medium-dose group > low-dose group > blank group. In summary, it can be demonstrated that the hair regrowth gel could promote the body hair regeneration and stimulate hair follicle growth in chemically depilated mice.

## 5. Materials and Methods

### 5.1. Reagents and Instruments

Isooctane, methanol, glycerol, propanediol, and triethanolamine solution are analytically pure (Chengdu Cologne Chemical Reagent Factory, Chengdu, China); laurocapram, carbomer (Shanghai McLean Biochemical Technology Co., Ltd., Shanghai, China); sodium chloride, KOH are solid reagents (analytically pure, Tianjin Aupu Sheng Chemical Co., Ltd., Tianjin, China); papain (purchased from Beijing Solepol Technology Co., Ltd., Beijing, China); AL104 electronic analytical balance (METTLER-TOLEDO Co., Ltd., Greifensee, Switzerland); RO50 laboratory (ultra-) pure water machine (Xiamen Keflon Ltd., Xiamen, China); Model H1650-W Centrifuge (Hunan Xiangyi Laboratory Instrument Development Co., Ltd., Xiangtan, China); Model KQ-250B Ultrasonic Cleaner (Kunshan Ultrasonic Instrument Co., Ltd., Kunshan, China); Model WGLL-16/12C Electric Heating Blast Thermostatic Drying Oven (Tianjin Taiste Instrument Co., Ltd., Tianjin, China); Model TGL-16 Controlled Temperature Adjustable Electric Furnace (Sichuan Shuke Instrument Co., Ltd., Chengdu, China); Model HH-S6A Electrothermal Constant Temperature Water Bath (Beijing Kewei Yongxing Instrument Co., Ltd., Beijing, China); KM mice, weighing 18–25 g (Chengdu Dashuo Laboratory Animal Co., Ltd., Chengdu, China); Evaporating Dish; Desiccator. The bear fat was sourced from Sichuan Dujiangyan Xinyuan Black Bear Breeding Co. (Dujingyan, China) (the bear fat used in the experiment was obtained after the natural death of the bears).

### 5.2. Quantitative Analysis of Bear Grease

According to the previous literature [13], the extraction rate (*η*, %) of bear grease could be calculated by Equation (1).

(1) $$\eta =\frac{A}{B}\times 100\%$$

where *A* and *B* denote the mass (g) of extracted bear oil and sample, respectively.

### 5.3. Enzymatic Hydrolysis Extraction Procedure

**Figure 9:**
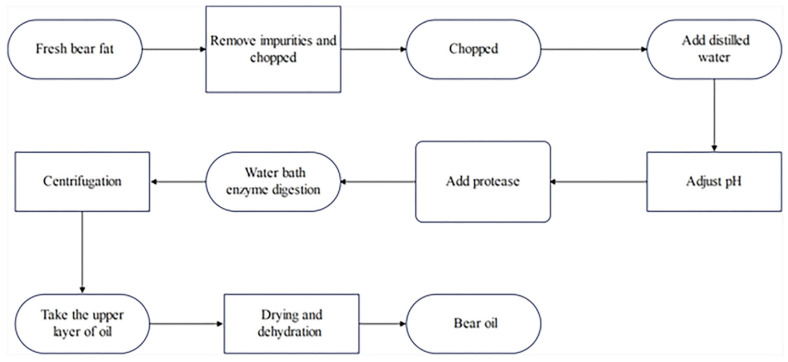
Experimental process of bear fat extraction.

In this study, the technical route is depicted in Figure 9.

### 5.4. Experimental Program of Bear Grease Extraction by Enzymatic Method

Here, the uniform experimental design method was used to optimize the extraction conditions, and the extraction rate of bear grease was used as the evaluation index, and the enzyme addition (1000, 2000, 3000, 4000, 5000, and 6000 U·g^−1^), the enzyme digestion temperature (50, 51, 52, 53, 54, and 55 °C), the enzyme digestion time (0.5, 1, 2, 3, 4, and 5 h), the pH (5.0, 5.5, 6.0, 6.5, and 7.0), the liquid to material ratio (1:1, 1:2, 1:4, 1:5, and 1:6) were selected as the major influential variables, and the experimental arrangement is shown in Table 4.

### 5.5. Fatty Acid Composition Analysis

#### 5.5.1. Methyl Esterification of Bear Resin

Accurately weigh 0.2 g of extracted bear grease, add isooctane to make it completely dissolved and volume to 10 mL, shake well, then take 50 μL in 10 mL test tube with pipette gun, and add 0.4 mol/L KOH–methanol solution 2.00 mL. Put it into a rotary oscillator for 2 min, put it into the test tube for 10 minutes, add 1.95 mL of isooctane, shake for 2 min, and then dilute it to 10 mL with sodium chloride solution with mass fraction of 8.0%. Centrifuge for 10 min at 2000 r/min and suck the supernatant into the micro test tube to make methyl esterification sample 10 mL; centrifuge at 2000 r/min for 10 min [14].

#### 5.5.2. GC-MS Analysis

The GC-MS analytical conditions were determined according to the relevant literature [15,16], and corresponding changes were made as follows: column: Agilent 19091S-433UI −60–325 °C 30 m × 250 μm × 0.25 μm; starting temperature 100 °C, held for 4 min, and then heated up to 250 degrees Celsius at 5 °C/min after a constant temperature of 16 min; bears lipids inlet temperature of 250 °C, the interface temperature of 250 °C; the carrier gas is helium, the flow rate of 1 mL/min, shunt ratio 10:1; EI source: electron energy 70 eV, ion inlet temperature 230 °C, quadrupole temperature 150 °C; standard tuning; SCAN mass scanning; solvent delay of 3 min; electron multiplier voltage of 1.635 V; scanning mass range of 10–550 amu.

### 5.6. Pharmacodynamic Pre-Experimental Methods

#### 5.6.1. Preparation of Gels

Next, 2% of Cb940 and 10% glycerol milled, add the appropriate amount of purified water and fully dissolve. Add 20% propylene glycol, 1% nitrone, 0.02% VE, and put forward to the bear grease, stirring well, using triethanolamine to adjust the pH to 7.0. Then, add the appropriate amount of purified water, stirring well to be obtained [17,18,19].

#### 5.6.2. Hair Growth Activity Test Method

KM mice aged 6–8 weeks were provided by Chengdu Dashuo Laboratory Animal Co., Ltd. (Chengdu, China) and were raised in a specific pathogen-free area under sterile conditions, with temperatures of 18–22 °C, humidity of 40–70%, and a normal diet, with free access to food. Animal experiments were conducted in accordance with the National Act on the Use of Experimental Animals (China) and approved by the Sichuan Committee on Laboratory Animals (approval number: SYXK2019-216). After 1 week of acclimatization, fifty mice of the Kunming breed were taken, and the depilatory cream was applied to the back of the mice for about 5 min and washed with warm water, so as to make the back of the mice smooth and free of injuries and residual hairs as net. The depilatory area was about 3 × 4 cm^2^. This operation was repeated until the mice entered the resting period of the hairs. After that, the mice were randomly divided into four groups of ten mice each, and blank matrix, 50%, 75%, and 100% gel (corresponding to the low-, medium-, and high-dose groups, respectively), was applied to the dehairing area twice a day, each time at 0.2 g/pupil, and the drug was administered for 15 consecutive days. On the 16th day, the mice were removed from the neck and executed. The hair removal area on the back was cut off, and a round piece of skin was taken from the same position of the hair removal area of each mouse with a 14 mm perforator. All the body hairs on the piece of skin were scraped off with a spatula, and the weight was weighed on a 1 in 10,000 balance to calculate the average hair weight of each group. Then, a skin slice of the same size was taken from the dehairing area of the mice, immersed in 4% paraformaldehyde solution and fixed for 24 h, and then routinely embedded in paraffin-embedded slices, hematoxylin–eosin staining (HE staining) with 3 consecutive slices in each case, and observed under a light microscope. The number of hair follicles in 3 fields of view was counted in each case, and the mean value was taken as the number of hair follicles in that case. The mean value of the number of hair follicles in each group of mice was found [20].

## 6. Conclusions

In this paper, the extraction process of bear grease was optimized by the uniform experimental design method, combined with the visual analysis of experimental data, and the optimal extraction process of bear grease was obtained in the following ranges: pH 6.5–7, extraction temperature 52–54 °C, liquid-to-feed ratio of 1:1–2:1, enzyme digestion time of 4.5–5 h, and enzyme additive amount of 5000–6000 U·g^−1^. After validation tests, the extraction rate of bear grease reached 81.89%, which was much higher than the average value, indicating that the optimized process was practical and reliable. Moreover, the bear grease extracted by the optimized process and by the traditional process was compared by GC-MS compositional analysis, and the results showed that the unsaturated and saturated fatty acids of the bear grease extracted by the optimized process were larger than those of the bear grease extracted by the traditional process. Pharmacodynamic pre-tests showed that bear grease has a good effect on hair growth and deserves further study.

There are few studies on bear grease, and its fatty acids and other components with medicinal value have not been explored. With the success of artificial breeding, it is valuable to explore the active ingredients and pharmacological effects of bear grease, which can provide certain data support for the development and utilization of bear grease and lay a good foundation for subsequent medicinal research.

## Figures and Tables

**Table 1:** Extraction rate of the bear grease by enzymatic method.

Groups	Extraction Rate (%)	Groups	Extraction Rate (%)
N1	47.27 ± 0.87	N16	7.33 ± 0.51
N2	19.53 ± 1.79	N17	7.20 ± 0.25
N3	71.54 ± 1.12	N18	23.97 ± 0.86
N4	17.17 ± 0.46	N19	74.59 ± 0.84
N5	75.64 ± 0.84	N20	57.63 ± 0.82
N6	5.11 ± 1.07	N21	63.66 ± 0.75
N7	76.12 ± 1.11	N22	89.37 ± 0.32
N8	63.89 ± 0.78	N23	57.2 ± 0.74
N9	42.42 ± 1.50	N24	87.78 ± 0.24
N10	19.2 ± 1.14	N25	19.31 ± 0.30
N11	74.74 ± 0.59	N26	20.00 ± 0.78
N12	19.18 ± 0.66	N27	42.64 ± 0.66
N13	82.37 ± 1.15	N28	14.24 ± 0.57
N14	69.86 ± 0.84	N29	21.90 ± 0.72
N15	33.08 ± 0.75	N30	16.15 ± 0.71

**Table 2:** GC-MS fatty acid class composition analysis of bear grease obtained by simmering method.

Serial No.	Name of Fatty Acid	Relative Content (%)
1	Pentadecanoic acid	1.16 ± 0.07
2	palmitoleic acid	9.80 ± 0.15
3	Palmitic acid	19.75 ± 0.41
4	(10*Z*)-10-Heptadecenoic acid	2.25 ± 0.17
5	Margaric acid	1.50 ± 0.35
6	9,12-Octadecadiynoic acid	16.76 ± 0.46
7	analytical standard	35.88 ± 0.73
8	*cis*-Vaccenic acid	3.73 ± 0.04
9	Arachidonic acid	0.33 ± 0.076
10	*cis*-13-Eicosenoic acid	0.46 ± 0.06
11	Adrenic Acid	0.61 ± 0.045

**Table 3:** GC-MS fatty acid class composition analysis of bear grease obtained by enzymatic digestion method.

Serial No.	Name of Fatty Acid	Relative Content (%)
1	Myristoleate	0.34 ± 0.050
2	Myristic acid	2.42 ± 0.38
3	Pentadecanoic acid	1.11 ± 0.16
4	palmitoleic acid	9.97 ± 0.23
5	Palmitic acid	19.7 ± 0.64
6	(10*Z*)-10-Heptadecenoic acid	2.31 ± 0.49
7	99,12-Octadecadiynoic acid	15.26 ± 0.29
8	analytical standard	36.99 ± 0.58
9	*cis*-Vaccenic acid	3.55 ± 0.28
10	Arachidonic acid	0.38 ± 0.059
11	*cis*-13-Eicosenoic acid	0.51 ± 0.085
12	Adrenic Acid	0.64 ± 0.075

**Table 4:** Uniform experimental design.

Groups	pH	Enzyme Digestion Time (h)	Enzyme Digestion Temperature (°C)	Liquid to Material Ratio	Enzyme Addition (U/g)
N1	5.5	5	54	6	4000
N2	5.5	3	55	4	1000
N3	5.5	1	51	1	1000
N4	6.5	1	50	3	6000
N5	7.0	2	54	2	1000
N6	7.0	1	53	5	2000
N7	6.5	3	52	2	3000
N8	5.0	5	51	3	2000
N9	5.5	2	53	3	2000
N10	6.0	0.5	50	6	2000
N11	5.5	4	50	2	5000
N12	6.0	2	51	4	1000
N13	7.0	0.5	51	1	4000
N14	5.0	4	52	4	5000
N15	6.5	0.5	54	4	4000
N16	6.5	2	51	5	5000
N17	5.0	1	53	6	3000
N18	6.0	3	52	1	4000
N19	5.0	2	54	1	6000
N20	7.0	5	50	4	3000
N21	7.0	4	53	3	5000
N22	6.0	5	53	2	6000
N23	6.5	4	52	6	1000
N24	6.5	5	55	1	2000
N25	6.0	1	55	3	5000
N26	5.0	3	50	5	4000
N27	6.0	4	54	5	3000
N28	7.0	3	55	6	6000
N29	5.0	0.5	55	2	3000
N30	5.5	0.5	52	5	6000

## Data Availability

Data are contained within the article.

## References

[1] Chen X., Li Z., Smith S.A., Chen M., Liu H., Zhang J., Tang L., Li J., Liu Q., Wu X. (2022). Optimization of Supercritical CO_2_ Extraction of Moringa Oleifera Seed Oil Using Response Surface Methodological Approach and Its Antioxidant Activity. Front. Nutr..

[2] Mwaurah P.W., Kumar S., Kumar N., Attkan A.K., Panghal A., Singh V.K., Garg M.K. (2019). Novel Oil Extraction Technologies: Process Conditions, Quality Parameters, and Optimization. Compr. Rev. Food Sci. Food Saf..

[3] Pinela J., Fuente B.D.L., Rodrigues M., Pires T.C., Mandim F., Almeida A., Dias M.I., Caleja C., Barros L. (2022). Upcycling Fish by-Products into Bioactive Fish Oil: The Suitability of Microwave-Assisted Extraction. Biomolecules.

[4] Ng Y.J., Tham P.E., Khoo K.S., Cheng C.K., Chew K.W., Show P.L. (2021). A Comprehensive Review on the Techniques for Coconut Oil Extraction and Its Application. Bioprocess Biosyst. Eng..

[5] Aitta E., Damerau A., Marsol-Vall A., Fabritius M., Pajunen L., Kortesniemi M., Yang B. (2023). Enzyme-Assisted Aqueous Extraction of Fish Oil from Baltic Herring (Clupea Harengus Membras) with Special Reference to Emulsion-Formation, Extraction Efficiency, and Composition of Crude Oil. Food Chem..

[6] Kumar S.P.J., Prasad S.R., Banerjee R., Agarwal D.K., Kulkarni K.S., Ramesh K.V. (2017). Green Solvents and Technologies for Oil Extraction from Oilseeds. Chem. Cent. J..

[7] Mat Yusoff M., Gordon M.H., Niranjan K. (2015). Aqueous Enzyme Assisted Oil Extraction from Oilseeds and emulsion De-Emulsifying Methods: A Review. Trends Food Sci. Technol..

[8] Wang Q.L., Jiang J., Li J.W., Qiu M.B., Lin C.Z., Shi X.H., Cao P.R., Liu Y.F. (2015). High Quality Lard with Low Cholesterol Content Produced by Aqueous Enzymatic Extraction and Β-Cyclodextrin Treatment. Eur. J. Lipid Sci. Technol..

[9] Sun Y., Yan J., Jia N., Zhong M., Zhai C. (2012). Visual Analysis of Extraction Process of Paprika Seeds Oil with Supercritical Carbon Dioxide. Food Sci. Technol..

[10] Zhou J., Wang M., Saraiva J.A., Martins A.P., Pinto C.A., Prieto M.A., Simal-Gandara J., Cao H., Xiao J., Barba F.J. (2022). Extraction of Lipids from Microalgae Using Classical and Innovative Approaches. Food Chem..

[11] Dejoye Tanzi C., Abert Vian M., Chemat F. (2013). New Procedure for Extraction of Algal Lipids from Wet Biomass: A Green Clean and Scalable Process. Bioresour. Technol..

[12] Alavijeh R.S., Karimi K., Wijffels R.H., van den Berg C., Eppink M. (2020). Combined Bead Milling and Enzymatic Hydrolysis for Efficient Fractionation of Lipids, Proteins, and Carbohydrates of Chlorella Vulgaris Microalgae. Bioresour. Technol..

[13] Xia Y.S., Sun Y.S., Liu C., Li Z.M., Ren D.D., Mu R., Zhang Y.T., Bo P.P., Zhao L.J., Wang Z. (2021). Effect of Aqueous Enzymatic Extraction of Deer Oil on Its Components and Its Protective Effect on Gastric Mucosa Injury. Front. Nutr..

[14] Li J., Zhang S., Gu X., Xie J., Zhu X., Wang Y., Shan T. (2022). Effects of Alfalfa Levels on Carcass Traits, Meat Quality, Fatty Acid Composition, Amino Acid Profile, and Gut Microflora Composition of Heigai Pigs. Front. Nutr..

[15] Deme T., Haki G.D., Retta N., Woldegiorgis A., Geleta M. (2021). Fatty Acid Profile, Total Phenolic Content, and Antioxidant Activity of Niger Seed (*Guizotia abyssinica*) and Linseed (*Linum usitatissimum*). Front. Nutr..

[16] Xu Z., Chen W., Wang L., Zhou Y., Nong Q., Valencak T.G., Wang Y., Xie J., Shan T. (2021). Cold Exposure Affects Lipid Metabolism, Fatty Acids Composition and Transcription in Pig Skeletal Muscle. Front. Physiol..

[17] Castro R.F., Azzalis L.A., Feder D., Perazzo F.F., Pereira E.C., Junqueira V.B.C., Rocha K.C., Machado C.D.A., Paschoal F.C., Gnann L.A. (2012). Safety and Efficacy Analysis of Liposomal Insulin-Like Growth Factor-1 in a Fluid Gel Formulation for Hair-Loss Treatment in a Hamster Model. Clin. Exp. Dermatol..

[18] Guan M., Chu G., Jin J., Liu C., Cheng L., Guo Y., Deng Z., Wang Y. (2022). A Combined Cyanine/Carbomer Gel Enhanced Photodynamic Antimicrobial Activity and Wound Healing. Nanomaterials.

[19] Osipitan O.O., Shi Y., Di Pasqua A.J. (2021). Phenethyl Isothiocyanate-Containing Carbomer Gel for Use against Squamous Cell Carcinoma. Pharmaceutics.

[20] Xia M.-Q., Tian C.-L., Liu L., Hu R.-F., Gui S.-Y., Chu X.-Q. (2020). Transdermal Administration of Ibuprofen-Loaded Gel: Preparation, Pharmacokinetic Profile, and Tissue Distribution. AAPS PharmSciTech.

